# Classification of single-trial auditory events using dry-wireless EEG during real and motion simulated flight

**DOI:** 10.3389/fnsys.2015.00011

**Published:** 2015-02-17

**Authors:** Daniel E. Callan, Gautier Durantin, Cengiz Terzibas

**Affiliations:** ^1^Center for Information and Neural Networks (CiNet), National Institute of Information and Communications Technology (NICT), Osaka UniversityOsaka, Japan; ^2^Multisensory Cognition and Computation Laboratory Universal Communication Research Institute, National Institute of Information and Communications TechnologyKyoto, Japan; ^3^Centre Aéronautique et Spatial, Institut Supérieur de l’Aéronautique et de l’Espace, Université de ToulouseToulouse, France

**Keywords:** EEG, dry EEG, brain machine interface, independent component analysis, Kalman filter, auditory evoked response, single trial, classification

## Abstract

Application of neuro-augmentation technology based on dry-wireless EEG may be considerably beneficial for aviation and space operations because of the inherent dangers involved. In this study we evaluate classification performance of perceptual events using a dry-wireless EEG system during motion platform based flight simulation and actual flight in an open cockpit biplane to determine if the system can be used in the presence of considerable environmental and physiological artifacts. A passive task involving 200 random auditory presentations of a chirp sound was used for evaluation. The advantage of this auditory task is that it does not interfere with the perceptual motor processes involved with piloting the plane. Classification was based on identifying the presentation of a chirp sound vs. silent periods. Evaluation of Independent component analysis (ICA) and Kalman filtering to enhance classification performance by extracting brain activity related to the auditory event from other non-task related brain activity and artifacts was assessed. The results of permutation testing revealed that single trial classification of presence or absence of an auditory event was significantly above chance for all conditions on a novel test set. The best performance could be achieved with both ICA and Kalman filtering relative to no processing: Platform Off (83.4% vs. 78.3%), Platform On (73.1% vs. 71.6%), Biplane Engine Off (81.1% vs. 77.4%), and Biplane Engine On (79.2% vs. 66.1%). This experiment demonstrates that dry-wireless EEG can be used in environments with considerable vibration, wind, acoustic noise, and physiological artifacts and achieve good single trial classification performance that is necessary for future successful application of neuro-augmentation technology based on brain-machine interfaces.

## Introduction

Technology capable of augmenting human performance by means of feedback of decoded neural states has potential for many types of neuroergonimic applications. Neuroergonomics is the study of the human brain in relation to performance at work, at home, in transportation, and in everyday settings with the goal of using this knowledge to design technologies and work environments to augment human behavior to enhance safety, usability, efficiency and enjoyment (Parasuraman, 2003; Parasuraman and Rizzo, 2008). Because of the inherent dangers in aviation and space operations these fields may be particularly well suited for neuroergonomic applications to increase performance and safety. There are many challenges for effective implementation of neuroergonomic technology in real-world situations. Unlike the laboratory where brain recordings can be made under controlled conditions, in real world situations there is considerable additional physiological and environmental noise that must be dealt with. In addition it is likely the case that brain dynamics differ in real-world environments compared to those of the laboratory (McDowell et al., 2013; Lin et al., 2014).

Pilots’ often face periods of excessive workload, stress, fatigue, attentional deficits, etc... during flight operations that may affect performance. The ability to decode the mental state of the pilot and augment these states by neuro- adaptive feedback and/or automation has great potential in improving training, performance, and safety. In this article we are particularly interested in investigating brain potentials that occur as a result of passive presentation of an auditory stimulus during flight. This research has relevance to the phenomenon of inattentional deafness in which pilots’ sometimes miss audio alarms. Missed audio alarms are responsible for a significant number of aviation accidents (Bliss, 2003; Scannella et al., 2013; Dehais et al., 2014).

There have been a number of studies in which brain activity has been measured with electroencephalography EEG during aviation (Sem-Jacobsen et al., 1959; Blanc et al., 1966; Caldwell and Lewis, 1995) and space operations (Maulsby, 1966; Cheron et al., 2009). EEG has been successfully collected in flight since the late 1950’s (Sem-Jacobsen et al., 1959). These studies showed that although the in flight environment is subject to noise caused by vibration and greater physical movement of the pilot that are not present during laboratory based experiments, the EEG signals recorded are able to show changes in various frequency bands and additional specific features that are associated with flight performance (Sterman et al., 1987) as well as workload (Howitt et al., 1978; Hankins and Wilson, 1998; Dussault et al., 2005) and fatigue (Howitt et al., 1978; Sauvet et al., 2014). Experiments conducted aboard the International Space Station have shown changes in the rhythmic brain activity of astronauts as a result of microgravity (Cheron et al., 2009). In an experiment conducted during parabolic flight it was found that a mental imagery (Thinking of moving arm vs. thinking of words) based brain machine interface (BMI) task could achieve between 72%–79% single trial classification performance across the various g-force conditions ranging from 0 to 2 g (Millàn Jdel et al., 2009).

A BMI is a device that provides for the ability to transfer and use information from distinct brain states for communicating with a machine (Blankertz et al., 2010). BMI based on electroencephalography EEG have been used in many applications including, but not limited to, modulating brain rhythms to control movement of a cursor on a screen (Wolpaw et al., 2002) as well as a quadrocopter in the real world (LaFleur et al., 2013), decoding brain states such as attention, performance capability, workload, etc… (Dornhege et al., 2007; Müller et al., 2008; Blankertz et al., 2010), decoding various perceptual events (Birbaumer et al., 1999; Wang et al., 2006; Bin et al., 2009). A primary goal of BMI research is to augment human behavior to allow for enhanced performance. Notably, there are considerable improvements in performance for some medical applications of BMI when the user’s motor system is severely incapacitated. For example patients with locked-in syndrome who are not able to communicate through standard pathways or assistive technology can learn to spell and initiate dialogs using P300 based BMI (Sellers et al., 2014). Non-medical application of the BMI P300 speller in normal healthy individuals is not practical since normal channels of communication are considerably better.

There are several limitations for the practical neuroergonomic application of many of these BMIs in real-world situations (Blankertz et al., 2010). Many of these BMIs take a considerable amount of time for operator training, on the order of months. This is true especially for implementations when the user needs to learn how to modulate various brain rhythms (Wolpaw et al., 2002; LaFleur et al., 2013). For practical application there should be very little if any operator training in control of the BMI. Many BMIs require considerable mental workload to operate, such as those requiring mental imagery and those utilizing focused attention away from the primary task environment. The amount of cognitive resources required to operate such BMI can be so high, that the operator’s usual channels of perceptual, motor, and cognitive processing are greatly impaired. For example, it is obvious that if one is actively involved with mentally imaging movement of their left hand to move a vehicle left, mentally imaging movement of both arms to move a vehicle right, mentally imaging walking to move forward, and mental word association to move back, that this will drastically compromise the operators ability to speak, walk, and move their hands while engaged in operating the BMI. However, if one uses a joystick to move a vehicle the operator will still be able to speak, walk, and move their hands around without drastically compromising performance depending on the difficulty of the task at hand. While P300 based BMI have advantages in that they do not require extensive subject training, they often require directed attention away from the environment in which they are suppose to operate in. For example subjects using a P300 based BMI to control various items in a virtual apartment reported a far lower sense of presence, which they attributed to increased workload, in interacting in the environment than did control subjects not using BMI (Groenegress et al., 2010). In order for a BMI to be effective it is necessary to utilize naturally occurring brain states for control. Another limitation of many of these BMI implementations is that they utilize rather bulky EEG systems that are not portable and use gel that takes a considerable amount of time and assistance to apply.

Practical application of EEG in real-world situations requires the use of technology that is easy to wear without the use of gel and one that is wireless to transmit data to a computer for real-time processing. There are many dry-wireless EEG systems available that have been used for many different applications including drowsiness detection (Park et al., 2011), gaming (Liao et al., 2012; Zao et al., 2014), and detection of perceptual events (Lin et al., 2014). Of particular interest in relation to real-world application of BMI is the study conducted by Lin et al. (2014) investigating the impact of walking locomotion on the ability to detect steady state visual evoked potentials (SSVEP) using a dry-wireless EEG system. The SSVEP is a frequency coded neural response that is modulated by the presentation frequency of the visual stimuli (Lin et al., 2014). The classification performance was good but decreased as a result of walking speed from 84.87% for standing to 83.03% for 1 MPH walking, 79.47% for 2 MPH walking, and 75.26% for 3 MPH walking (Lin et al., 2014). The decrease in performance as a function of walking speed may result from increasingly larger artifacts in the EEG as well as to greater difficulty in fixated attention to the visual stimuli as one moves more. Dry electrodes are more susceptible to movement related artifacts than gel based EEG recording (Guger et al., 2012; Lin et al., 2014). One limitation for utilization of the SSVEP for many BMI applications is that the task requires the subject to attend to the visual stimuli presented on the computer screen. This requirement is not practical in many real-world situations where perceptual motor control requires the use of the visual system such as in aviation and space operations.

The goal of this study is to determine the extent to which perceptually evoked brain related EEG signals, recorded with a dry-wireless system in noisy real-world environments can be decoded for potential use in BMIs to augment human performance. With a focus on aviation related applications we conducted our experiment in two environmental conditions. One environmental condition was the use of a motion platform based flight simulator the other was an open cockpit biplane. In order to avoid practical problems in controlling the airplane by using a visual based perceptual presentation task, an auditory stimulus presentation task was used to assess single trial classification performance. The advantage of using an audio stimulus presentation task (100 ms chirp sound) is that it can be played passively in the background while the pilot attends to flying the plane. The goal for the classifier was to determine from the EEG data the presentation of the audio stimulus (chirp) from periods of no audio stimulus (silence) presentation.

A control condition was used for both environmental conditions. In the motion platform condition the experiment was conducted with the platform and flight simulator off (a relatively noise free condition) and with the motion platform on while flying aerobatic maneuvers in the flight simulator (Platform Off and Platform On conditions). In the platform on condition there is considerable movement of the individuals arms, legs, eyes, neck, and entire body as well as potential electrical noise from the motion platform motors. In the biplane condition the experiment was conducted with the engine and avionics off while sitting on the tarmac (a relatively noise free condition) and with the engine and avionics on while piloting the plane in cruise flight. The open cockpit biplane may constitute one of the most challenging environments for EEG recording in which there is an incredible amount of vibration, wind, acoustic noise, as well as considerable movement of the individual’s arms, legs, eyes, neck, and entire body.

There are considerable sources of noise that make recording of EEG in real-world environments challenging including non-neurological electrophysiological signals (e.g., muscle activity), electronic noise, and mechanical vibrations. In order to be able to clean the data of the artifacts and extract brain activity related to the auditory evoked responses several procedures within EEGLAB (Swartz Center for Computational Neuroscience, Delorme and Makeig, 2004) are used including independent component analysis (ICA). In addition to these artifact-cleaning procedures a Kalman (1960) filter implementing a dynamical model of the single-trial auditory evoked responses was also used to help extract brain activity related to presentation of the audio stimuli. Based on the results of Lin et al. (2014) using a similar dry-wireless EEG system it is predicted that above chance classification performance in detecting single trial audio stimulus presentation will be achieved in all conditions. Furthermore, it is predicted that both ICA and Kalman filtering will improve classification performance across all conditions.

## Methods

### Subjects

The same subject was used for all studies. The subject was male, right handed, 45 years old, with normal hearing. One of the authors served as the subject in this experiment. He has 5 years of flying experience with more than 250 h total time and 200 h in biplanes of the same make and model. The subject gave informed consent for experimental procedures approved by the ethics committee of the National Institute of Information and Communications Technology in accordance with the principles expressed in the Declaration of Helsinki.

### Procedure

The experimental task consisted of passively listening to a chirp sound (0.1 s duration) in different environmental settings while recording brain activity using the Cognionics 64 channel dry wireless EEG system. The chirp sound was presented 200 times spaced randomly by at least 0.6–2.5 s of silence. Within these periods of silence 200 independent 0.5 s segments were randomly extracted. The different environmental settings and conditions are as follows: Electric Motion Platform (Platform Off, Platform On) and open cockpit Biplane (Engine Off, Engine On).

**Figure 1:**
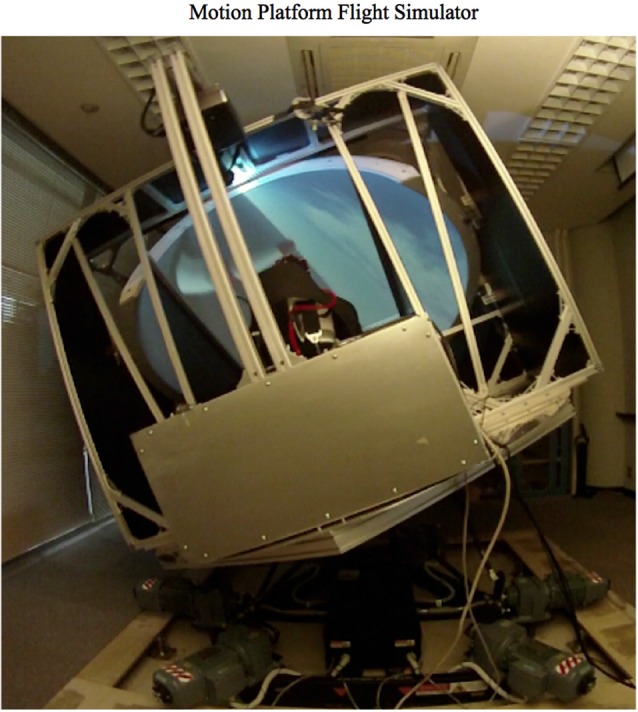
**Motion Platform Flight Simulator composed of the CKAS V7 6 degree of freedom Motion System (CKAS Mechatronics, Melborn, Australia) with a custom built cockpit utilizing dome projection for video**. The flight controls consist of 1. A control stick to manipulate the elevator (pitch) and ailerons (roll), 2. Rudder pedals to manipulate the rudder (yaw), and 3. A Throttle to manipulate thrust. Single-trial auditory events using dry-wireless EEG were evaluated while the subject was flying through a simulated Redbull Air Race course.

The CKAS V7 6 degree of freedom Motion System (CKAS Mechatronics, Melborn, Australia) with a custom built cockpit utilizing dome projection for video was used (see Figure 1). In the motors off condition the subject was sitting still in the chair mounted on the platform. In the motors on condition the subject was engaging in aerobatic flight simulation (X-Plane Laminar Research) through the same Redbull Air Race course as used in a previous fMRI study (Callan et al., 2012). See Callan et al. (2012, 2013) for details on the implementation of the flight simulator system for brain imaging experiments. Just as in a real airplane, the subject controlled the ailerons and elevator by stick with the right hand, the throttle lever with the left hand, and the rudders with foot pedals. The motors of the motion platform moved in relation to the accelerations of the aircraft in the flight simulator. No sound was presented from the flight simulator. In both the platform off and platform on conditions the audio stimuli were presented using the Clarity Aloft Pro aviation headset using the same sound level. The experiment took approximately 10 min for each condition. The platform on condition was conducted first followed by the platform off condition.

**Figure 2:**
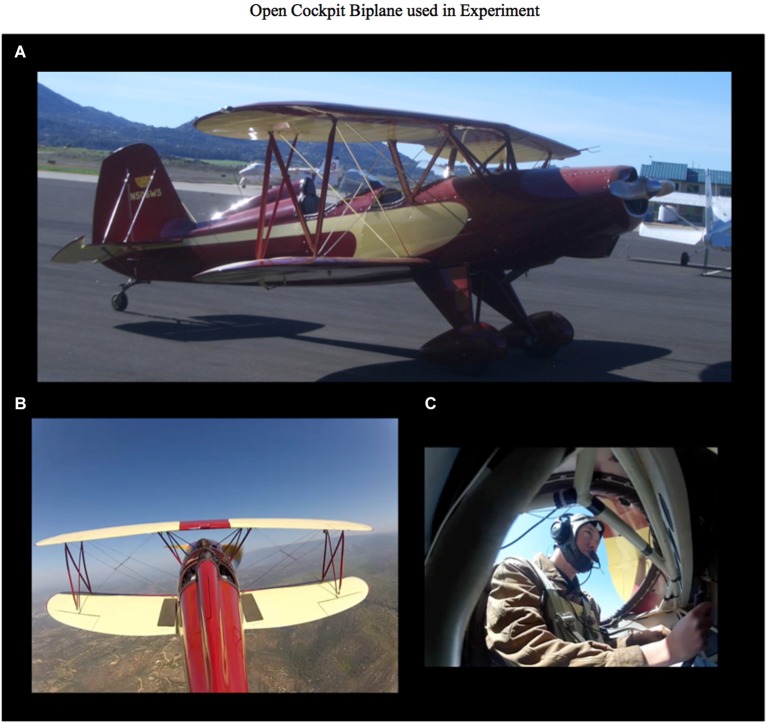
**Open Cockpit Starduster SA300 Biplane used for in flight and ground testing of single-trial auditory events using dry-wireless EEG. (A)** View off the biplane on the ground. **(B)** View of the biplane in the air. **(C)** Picture of the subject wearing the Cognionics 64 channel dry-wireless EEG under the leather flight helmet. This is the same EEG system pictured in Figure 3.

The biplane used in the experiment was an open cockpit two passenger Starduster SA300 (see Figure 2). In the engine off condition the subject was sitting in the front seat of the biplane on the tarmac with avionics off. In the engine on condition the subject was piloting the plane during cruise flight from the front seat with avionics on. In the engine on condition there was extreme vibration, acoustic noise, and wind. In both the engine off and engine on conditions the Cognionics EEG system was worn underneath a leather flight cap. The audio stimuli were presented using the Bose A20 active noise canceling aviation headset using the same sound level. The experiment took approximately 10 min for each condition. The engine off condition was conducted first followed by the engine on condition.

**Figure 3:**
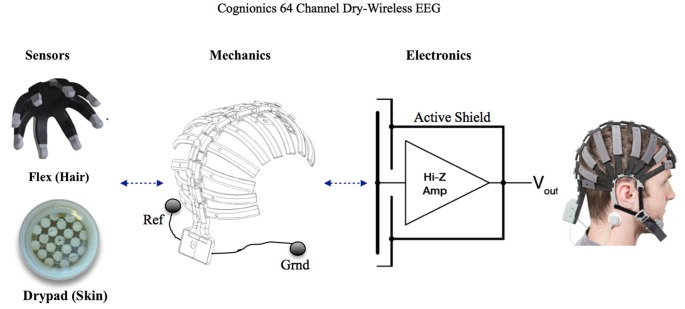
**Cognionics 64 Channel Dry-Wireless EEG Headset**. Two types of dry sensors are used: The flex sensors are placed over hair and the Drypad sensors are laced over bearskin, such as the forehead. In our setup, we utilized 7 Drypad sensors on the forehead (first band) and 54 flex sensors across the rest of the array. Each band of the headset is user adjustable to enable proper fit and sensor pressure over different head sizes and shapes. An internal flexible conductive layer forms an active shield, which spans the entire headset, to minimize external noise pickup even with high-impedance dry electrodes. Ground (Grnd) and reference (Ref) electrodes are placed under the ear as shown in the figure. The same cap pictured here is in Figure 2 underneath the leather flight helmet. See the text in the Methods section for further details.

### EEG recording and analysis

EEG was recorded using the Cognionics HD-72 dry wireless EEG headset (Cognionics, Inc., San Diego^1^). The same 64-channel EEG system was used by Mullen et al. (2013) and shares the same underlying technology as the 32-channel system reported in Chi et al. (2013) and Lin et al. (2014).

The EEG headset consists of a mechanically flexible spine to provide structure and ease of handling. Each segment of the spine contains a row of electrodes. The 64 electrodes provide full scalp coverage. An internal active shield, covering all sensor positions, spans the entire headset to minimize external noise pickup and artifacts. Reference and ground are placed on the mastoids with by two standard ECG adhesive electrodes (see Figure 3).

Two sensor options are compatible with the headset and connect via a miniature snap receptacle. In the setup, seven Drypad sensors were placed on the forehead (Figure 3). The Drypad electrode is a cushioned membrane that is optimized for bare skin contact. For the remaining 57 positions, we used the Flex sensors designed for thru-hair measurements. The Flex sensor is specifically designed to brush aside hair and make direct scalp contact with legs that gently bend and deform under modest pressure. Compared to previous metal pin sensors (e.g., Liao et al., 2011), the Flex sensors offer improved comfort and safety since the sensors can completely flatten under hard impacts. Contact impedances with both sensors typically range from 100 k to 1 MOhm (Mullen et al., 2013) on unprepared skin.

To adequately acquire EEG signals with high electrode impedances, the Cognionics system utilizes a combination of active shielding, high input impedance amplifiers and active grounding to cancel and minimize environmental noise. Data is sampled at 300 Hz (DC-80 Hz bandwidth) with 24-bit ADCs to maximize dynamic range and quickly recover from overload artifacts. Total noise within the EEG band (1–50 Hz) is 0.7 microVolts RMS. The signal quality of the sensors, electrodes, and the data acquisition circuitry has been shown to be comparable to wet electrode based EEG systems with a correlation between simultaneously recorded evoked potentials of *r* > 0.9 (Chi et al., 2013; Mullen et al., 2013).

All electronics are housed in a miniature box at the base of the headset, which weighs a total of only 350 g making the entire system lightweight, portable and wearable. Data transmission is accomplished by a custom 5 GHz WiFi module. The headset radio communicates directly to an embedded WiFi host, in a point-to-point network, running on a USB dongle to minimize the overhead associated with a typical access point and PC-based software protocol stack, thereby minimizing latency and jitter. The receiving computer was a Panasonic CF-AX2 running Ubuntu Linux operating system. The average delay in receiving the signal was less than 1 ms. Audio stimuli were presented from the same computer as used to record the EEG by means of the auxiliary input to the aviation headset. The presentation of the audio signals was given time stamps in correspondence with the time stamps given for each of the recorded EEG samples. The high temporal precision of the wireless link and the presentation software allows for accurate time registration between stimulus and EEG without the use of wire-line markers.

The EEG data was preprocessed using artifact-cleaning procedures available in EEGLAB (Delorme and Makeig, 2004). The data was first filtered using a FIR band pass filter between 2 and 30 Hz with a filter order of 496. The data was separated into a training set of 75% of the stimuli (150 chirp stimuli and 150 silent stimuli) and a testing set of 25% of the stimuli (50 chirp stimuli and 50 silent stimuli) for the platform off and on conditions and the biplane engine off and on conditions. The rational for choosing 75% of the trials for training and 25% for testing is that we believed this division would provide enough training data to generalize to a novel testing set while maintaining enough trials in the testing set to be a reasonable representation of the population. Although we did not attempt to train and test on a 50% split of the data we believed that the amount of training data may not have been sufficient for generalization to the novel test set. The artifact cleaning procedures were applied only to the training sets. The results of which were then applied to the corresponding test sets. The exact same cleaning procedures were then applied to the corresponding testing sets, with the same parameters.

Channels were considered to be bad and were removed from the training data based on the following: Maximum amplitude in the channel is greater than 100 microvolts; Channel flatline duration is greater than 5 s; Channel is correlated at less than 0.8 to its robust estimate based on other channels; Channel has more line noise relative to its signal than 4 standard deviations from the channel population mean. These same channels were then removed from the corresponding test sets.

The next step was to clean the multi-channel training data with the artifact subspace reconstruction (ASR) method using the defaults given in the clean_artifacts software within EEGLAB. ASR allows for the removal of non-stationary high-variance signals from EEG and reconstructs missing data using a spatial mixing matrix (See EEGLAB software by Christian Kothe and the following for details^2^). Calibration data from clean segments of the training data were used to determine the ASR filters separately for each of the four conditions. The ASR filter was then applied to the corresponding test data.

Following cleaning of the data by ASR, ICA using the extended infomax algorithm (Bell and Sejnowski, 1995) in EEGLAB was applied in order to separate brain activity related to the auditory evoked potentials from other brain and artifact related components. The weights of the ICA were determined only from the training data separately for each of the four conditions (platform off, platform on, engine off, engine on). These weights were then applied to the corresponding test data to determine the ICA activation waveforms. See Figure 5 for an example of the continuous data before ASR, after ASR, and after ICA.

**Figure 4:**
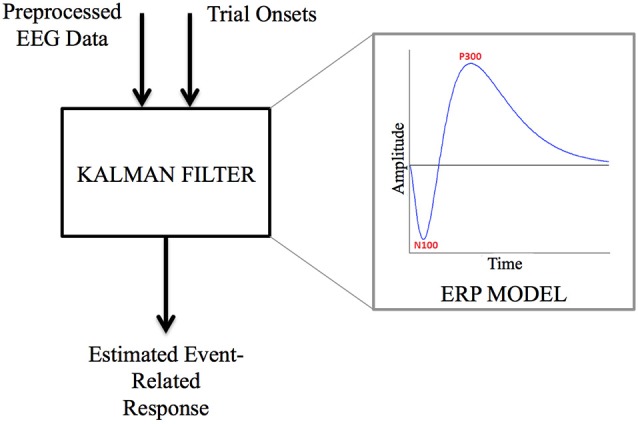
**Kalman filter functional model**. Kalman filter is designed from an Event Related Potential (ERP) dynamical model (on the right), and uses both preprocessed EEG data and trial onsets to estimate the instantaneous event-related response.

**Figure 5:**
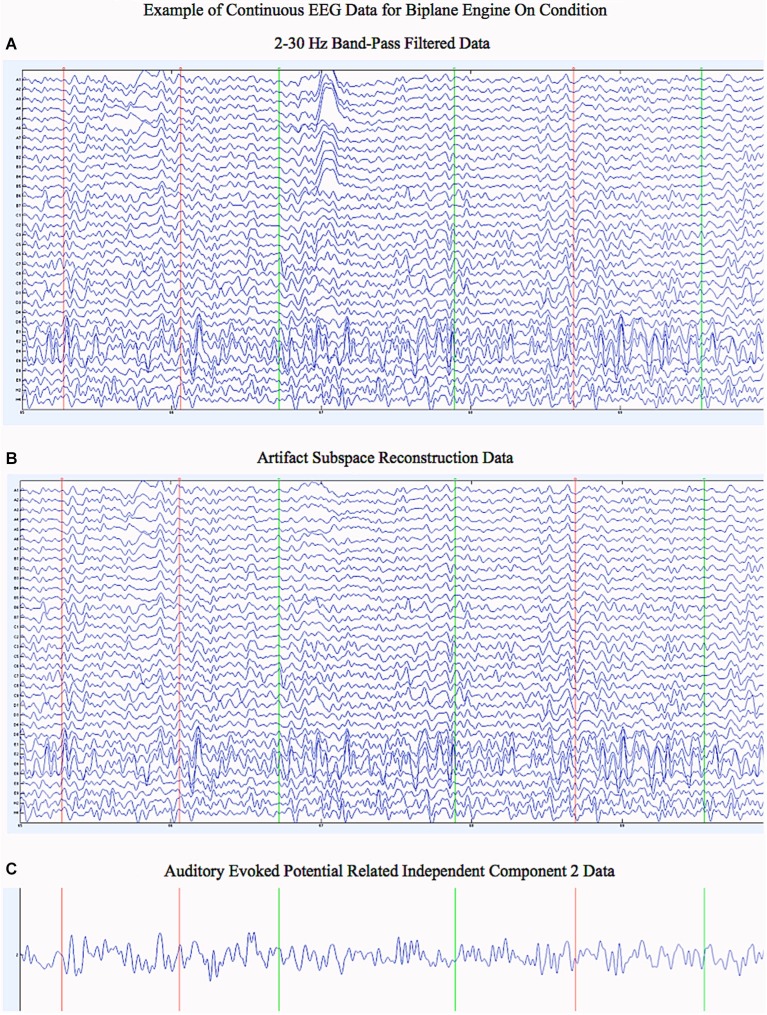
**Example of Continuous EEG Data for the Biplane Engine On Condition. (A)** Five seconds of continuous data from the test set that has been band pass filtered from 2–30 Hz for the 34 electrodes included. **(B)** Five seconds of continuous data from the test set that has undergone artifact subspace reconstruction as well as band pass filtered from 2–30 Hz for the 34 electrodes included. Notice how ASR has cleaned up the artifact present in many of the channels (compare with **(A)** above). **(C)** Five seconds of continuous data from the test set for auditory evoked potential related independent component number 2. The ICA was carried out over the data in **(B)**. Green lines denote the onset of audio stimuli and Red lines denote the onset of silent trials.

Kalman filtering (Kalman, 1960) was applied to the ASR filtered data both before and after ICA, to produce an estimate of the single-trial auditory evoked response. Kalman filtering is a linear quadratic estimation method, which relies on both the measurements and a modelization of the Event-Related Potential (ERP) dynamics to product an estimate in real-time. This method has been successfully applied to single-trial event related potentials estimation (Georgiadis et al., 2005). In our case, the parameters used as a model for the Kalman filter design were the N100 and P300 waves, generic features of the auditory evoked potential (see Figure 4). Both waves were modeled by a third-order impulse response with peaks respectively at 100 ms and 300 ms. The state noise was considered as white noise (Georgiadis et al., 2005), and the measurement noise covariance to state noise covariance ratio was fixed to 0.001, so that the Kalman estimate would put confidence in the measurements (Grewal and Andrews, 2008).

### Training and testing the classifier

The Matlab Least Squares Probabilistic Classification (LSPC) toolbox (Sugiyama, 2010) was used to determine how well single trial audio presented stimuli could be identified in the EEG signal from periods of audio silence. LSPC uses a linear combination of kernel function to model the class-posterior probability. Regularized least-squares fitting of the true class-posterior probability is used to learn its parameters (Sugiyama, 2010). The use of least-squares fitting to determine a linear model allows for a global solution to be made analytically providing a considerable speedup in computational time. The default parameters were used in training of the LSPC models (see Matlab code: Sugiyama, 2010).

The features used to train the classifier consisted of the samples from onset to 500 ms after stimulus onset (150 samples) for both chirp and silent stimuli. In the case of ICA data the components selected to be included in the LSPC model were determined by visual inspection of the mean of the activation waveform of the chirp training data stimuli that showed a characteristic auditory evoked potential. For the pre-ICA data all of the channels were included in the LSPC model. The large number of features relative to the number of trials used to train the classifiers has the potential for over-fitting, resulting in poor generalization to the novel test data. We utilized inner cross-validation procedures for model selection in part to assess and protect against potential over-fitting.

The following inner cross-validation procedures were used for model selection and testing: Randomized ten fold cross-validation was used on the training data and the trained model with the best performance was selected for evaluating performance on the test data. One fold of the training data consisted of 135 chirp and 135 silent stimuli and one fold upon which the model was evaluated for selection consisted of 15 chirp and 15 silent stimuli. The final test data consisted of 50 chirp and 50 silent stimuli. This procedure was conducted 100 times to determine the distribution of performance of the model given random aspects of training (e.g., the trials selected for training and their order into the model).

Several conditions were evaluated to assess the efficacy of ICA and Kalman filtering in improving classification performance on the data. These conditions included the four environmental conditions (Platform Off, Platform On, biplane Engine Off, biplane Engine On) and four preprocessing conditions (pre-ICA constituting the baseline no processing condition, ICA, pre-ICA Kalman filtered, ICA Kalman filtered) for a total of 16 analyses.

The statistical significance between the various conditions was determined by repeating the nested cross-validation process ten times for each condition. The nested cross-validation procedure is commonly used to estimate the stability of the model across conditions (Shenoy et al., 2008). At each step, we computed the classification accuracy, false positive rate and false negative rate. The differences between the classification results obtained from each of the preprocessing conditions were then compared using Wilcoxon sign-rank test.

## Results

The number of channels not rejected out of the 64 by the preprocessing steps for the various conditions are as follows: Platform Off (46), Platform On (20), Biplane Engine Off (30), Biplane Engine On (34). The independent components showing auditory evoked potentials used to train the LSPC models were the following for the various conditions: Platform Off (2, 3), Platform On (1, 3), Biplane Engine Off (2, 3), Biplane Engine On (2, 16). The components are sorted in descending order of mean projected variance. Therefore, the lower the component number the larger is the projected variance. Parentheses.

The results of the single trial classification performance for audio chirp verses silent stimuli for all environmental and analysis method conditions were significantly above chance (*p* < 0.05) based on permutation testing of 100 random shuffling of the labels compared to the mean performance of the respective models trained with correct labels. The accuracy, false positive rate, false negative rate, and d’ (sensitivity) along with the standard errors in parentheses for the platform off and platform on conditions for the four analysis conditions (Pre-ICA, ICA Pre-ICA + Kalman Filter, and ICA + Kalman Filter) are given in Table 1. The corresponding results for the biplane Engine Off and Engine On conditions are given in Table 2. The statistical significance of model stability for accuracy on the contrasts of pre-ICA vs. ICA, Off vs. On, and No Kalman filter vs. Kalman filter are given in Tables 3A–C. Testing for significance based on the 100 trials of the test set across the various contrasts using the Wilcoxon sign-rank test is given in Tables 4A–D.

**Table 1:** **Classification performance for the platform condition on the test data**.

	Platform off				Platform on			
	Accuracy (%)	False positive rate (%)	False negative rate (%)	d’	Accuracy (%)	False positive rate (%)	False negative rate (%)	d’
Pre-ICA	78.3 (0.33)	19.8 (0.20)	23.6 (0.65)	1.69 (0.01)	71.6 (0.56)	35.4 (1.52)	21.4 (0.60)	0.75 (0.08)
ICA	81.0 (0.49)	21.4 (0.99)	16.6 (0.73)	1.59 (0.07)	73.7 (0.52)	26.8 (1.91)	25.8 (1.75)	1.26 (0.12)
Pre-ICA + Kalman Filter	77.8 (0.55)	20.2 (1.05)	24.2 (1.94)	1.68 (0.07)	72.8 (0.53)	36.6 (1.58)	17.8 (1.94)	0.69 (0.08)
ICA + Kalman Filter	83.4 (0.27)	17.2 (0.53)	16.0 (0.60)	1.90 (0.04)	73.1 (0.8)	28.4 (2.61)	25.4 (1.79)	1.18 (0.16)

*The standard error is given in parentheses below each mean value of the 10 nested cross-validation iterations*.

**Table 2:** **Classification performance for the biplane condition on the test data**.

	Biplane engine off				Biplane engine on			
	Accuracy	False positive rate	False negative rate	d’	Accuracy	False positive rate	False negative rate	d’
Pre-ICA	77.4 (1.01)	20.2 (2.72)	25.0 (2.20)	1.75 (0.21)	66.1 (0.75)	38.0 (1.23)	29.8 (1.31)	0.61 (0.06)
ICA	78.5 (0.72)	20.0 (1.58)	23.0 (0.68)	1.71 (0.11)	77.3 (0.45)	23.6 (1.26)	21.8 (0.81)	1.45 (0.08)
Pre-ICA + Kalman Filter	77.0 (0.97)	14.6 (1.16)	31.4 (2.53)	2.13 (0.1)	65.3 (0.96)	35.4 (2.78)	34.0 (2.40)	0.78 (0.17)
ICA + Kalman Filter	81.1 (0.35)	10.0 (1.19)	27.8 (1.35)	2.65 (0.18)	79.2 (0.39)	22.6 (1.12)	19.0 (1.16)	1.51 (0.07)

*The standard error is given in parentheses below each mean value of the 10 nested cross-validation iterations*.

**Table 3:** **Statistical significance of the various contrasts using nested cross-validation to evaluate model stability**.

A. ICA vs. Baseline Pre-ICA
Overall	Platoff	Platon	Engoff	Engon	Platoff + Kalman	Platon + Kalman	Engoff + Kalman	Engon + Kalman
***		**		**		**		**
B. Off vs. On
Overall	Platform Pre-ICA	Engine Pre-ICA	Platform Pre-ICA + Kalman	Engine Pre-ICA + Kalman	Platform ICA	Engine ICA	Platform ICA + Kalman	Engine ICA + Kalman
***	**	**	**	**	**		**	*
C. Kalman Filter vs. no Kalman Filter
Overall	Platform Off Pre-ICA	Platform On Pre-ICA	Engine Off Pre-ICA	Engine On On Pre-ICA	Platform Off ICA	Platform On ICA	Engine Off ICA	Engine On ICA
*					**		*	**

Significance (one-tailed) is determined using the Wilcoxon sign-rank test. * = p < 0.05; ** = p < 0.01; *** = p < 0.001.

**Table 4:** **Statistical significance evaluated across trials of the test set for the various contrasts**.

A. ICA vs. Baseline Pre-ICA
Overall	Platform	Biplane	Platoff	Platon	Engoff	Engon
**		**				**
B. ICA + Kalman vs. Baseline Pre-ICA
Overall	Platform	Biplane	Platoff	Platon	Engoff	Engon
**		**			*	**
C. Off vs. On
Overall	Platform	Biplane
***	**	*
D. ICA + Kalman vs. ICA
Overall	Platform	Biplane	Platoff	Platon	Engoff	Engon
		*			*

Significance (one-tailed) is determined using the Wilcoxon sign-rank test. * = p < 0.05; ** = p < 0.01; *** = p < 0.001.

## Discussion

The primary objective of this experiment was to show as a proof of concept that a dry-wireless EEG system could be used in extremely noisy real-world environments while the operator is carrying out complex perceptual motor tasks and still show good classification performance of a perceptual event. Both the motion platform based flight simulation and the open cockpit biplane environments produced considerable artifacts challenging the ability for accurate classification of a perceptual event from EEG data. In the platform on condition the subject was flying through a Redbull Air Race course making abrupt banks to go through the cones in the correct orientation (Callan et al., 2013). The movement of the platform and the head and body to these banks produces large artifacts in the EEG trace. In the open cockpit biplane there is considerable vibration, wind, acoustic noise, and physiological noise resulting from control of the plane and movement of the body that produces large artifacts in the EEG trace.

Despite these challenging environments our study showed that far above chance classification performance can be achieved for all environmental conditions (Platform Off, Platform On, Biplane Engine Off, Biplane Engine On) and for all analysis conditions (Pre-ICA, Pre-ICA Kalman filtered, ICA, and ICA Kalman filtered) (See Tables 1–4). The classification performance was the best when applying both ICA and Kalman filtering to the data. This was especially true in the Biplane Engine On condition in which classification performance improved from 66.1% in the baseline pre-ICA no processing condition to 79.2% in the ICA Kalman filtering condition (See Tables 2–4). This classification performance was comparable to that of the Engine Off condition (81.1%) in which the biplane was sitting on the tarmac. It is unclear why the Platform On condition did not show the same beneficial effects of ICA Kalman filtering vs. the baseline no processing condition (73.1% vs. 71.6% respectively), as did the Biplane Engine On condition (See Tables 1–4). One possibility is that the abrupt movement of the body and head during the flight task produced non-stationary artifacts that could not be separated by the EEGLAB artifact cleaning methods or by ICA and Kalman filtering. In the future utilizing data from an accelerometer mounted on the head as a regressor of non-interest may be able to extract possible movement related artifacts and improve classification performance. The significantly greater accuracy for the Off over the On conditions is to be expected (See Tables 1–4) not only because of the much greater artifacts present in the On conditions but also because auditory responses are decreased depending on attention (Paul et al., 2014). It is likely that the attention needed for piloting in the On conditions reduced attention to the auditory stimuli.

The use of Kalman filtering was most effective when conducted on ICA processed data. Kalman filtering on pre-ICA data did not show improvement in performance compared to that of ICA processed data (See Tables 1–4). One possible explanation for these results is that ICA is able to separate the brain activity related to the auditory event from other brain activity and artifacts allowing for better estimation by the Kalman filter that utilizes generic information about the auditory evoked response as a dynamical model. In the pre-ICA data the brain activity for various processes as well as artifacts are mixed within all the channels and estimation of the auditory events by the Kalman filter model may be more difficult for these signals.

Because the results are only of a single subject it is not clear how well they generalize to other individuals. It is likely that different individuals with different levels of flight experience may show more/or less movement and physiological artifacts that may decrease/increase classification performance. The Cognionics dry wireless EEG system has already been shown to give good classification performance on a SSVEP test across a number of subjects (Lin et al., 2014). The Lin et al. (2014) study demonstrates that the Cognionics EEG system is able to extract from a group of individuals and is not just limited to a few special individuals. ICA has additionally been shown to be very good at extracting artifacts and task related components from EEG data (Delorme et al., 2007). This was also demonstrated for the data collected in this study and it is unlikely that it is only applicable to the single subject that participated. Despite these limitations the results do stand as a demonstration that it is possible to collect data in real-world environments that have considerable noise, both environmental and physiological, using dry-wireless EEG and obtain classification performance sufficient for some BMI augmentive neuroscience based applications. The research presented here lays a foundation for future aviation based studies where subject variability in EEG responses can be evaluated.

For effective use in implementing BMI in real-world applications it is necessary that the processes used for classification be fast enough for online control either in terms of manipulating robotic devices, giving feedback to the operator, and/or utilizing adaptive automation to augment human performance (Byrne and Parasuraman, 1996; Wilson and Russell, 2007). One of the key elements of this study was that the training set came from the first 75% of the data and the novel test set came from the last 25% of the data. All of the artifact-cleaning methods were applied to the training set and the same criteria and weights were then applied to the testing set. ASR, ICA, and LSPC classifier model selection by cross-validation requires some processing time depending on computer speed. In our case running the programs in Matlab on a MacBook Pro took around 10 min. The largest portion of time was spent computing the ICA. Therefore, implementation of a BMI would require a delay of approximately 10 min after the training data is collected before it could be applied if ICA is used. Just a couple minutes for model selection and training are required if ICA is not used. The extraction of channels, the ASR model, and the ICA weights from the training data as well as the Kalman filter can be applied without delay (the delay is the same as the sampling rate) to the test set. In this case the features used in the classification model were samples for 500 ms after stimulus onset. Therefore, depending on the type of initial band-pass filtering, the processing delay to control the BMI could be around 515 ms. Although the initial band-pass filter order for processing this data set was 496 samples, which would create a considerable delay it is possible to implement different types of filters with a lower order to decrease the processing delay. A delay of less than a second could certainly be used in a BMI to give a warning signal to the pilot or engage adaptive automation (Byrne and Parasuraman, 1996; Wilson and Russell, 2007), however, it is not sufficient for closed-loop control.

Given the significant number of aviation accidents that result from missed auditory alarms (Bliss, 2003; Scannella et al., 2013; Dehais et al., 2014), a BMI based on detection of auditory events could be quite important in giving alternate forms of feedback to warn the pilot when necessary. It is well known that high workload decreases the ability to perceive task-irrelevant and/or unattended visual and auditory information (Lavie, 1995, 2005, 2010). This phenomenon is called inattentional blindness with respect to vision (Mack and Rock, 1998) and inattentional deafness with respect to audition (Wood and Cowan, 1995; Macdonald and Lavie, 2011; Dehais et al., 2014). Inattentional deafness is known to be a critical factor in missing of auditory alarms in aeronautics (Dehais et al., 2014). It is interesting that although task irrelevant auditory stimuli can be presented in the background without any decrement in task related performance, the evoked brain potentials to these auditory events is modulated by workload on the primary task (Krammer et al., 1995). As such, it may be possible to assess the workload of the pilot and likely occurrences of inattentional deafness by investigating brain response to auditory events. An EEG based BMI system could potentially be used to decode the mental state of the pilot for occurrence of inattantional deafness and augment their mental state by feedback through a different modality or engage adaptive automation. Considerably more research needs to be conducted to determine on a single event basis whether an incidence of inattentional deafness is present or not before such a system can be implemented.

When one considers that brain dynamics are different while undergoing complex tasks in natural environments from that of the laboratory (McDowell et al., 2013; Lin et al., 2014) and that dry EEG systems are susceptible to movement-based artifacts (Guger et al., 2012; Lin et al., 2014) the single-trial classification performance of auditory perceptual events by a dry-wireless EEG system in the robust flight conditions of our study are quite impressive. This research demonstrates the feasibility of new avenues for investigating brain processes in real-world situations that cannot be conducted in the laboratory. In addition the research presented here also demonstrates that plausible BMI using dry-wireless EEG can be implemented in real-world settings to augment human performance.

## Conflict of interest statement

The authors declare that the research was conducted in the absence of any commercial or financial relationships that could be construed as a potential conflict of interest.

## Footnotes

^1^http://www.cognionics.com/

^2^http://sccn.ucsd.edu/eeglab/plugins/ASR.pdf
